# Attempted Suicides in the Elderly With Existing DNRs: An Emerging Geriatric Ethical Dilemma

**DOI:** 10.7759/cureus.12658

**Published:** 2021-01-12

**Authors:** Kelvin Tran, Pauline Chen, Tessy Korah

**Affiliations:** 1 Psychiatry, University of Florida, Gainesville, USA; 2 Psychiatry, University of Florida, Gainsville, USA

**Keywords:** psychiatry, clinical, geriatrics, suicide, pact, depression, ethics, autonomy, beneficence, dnr

## Abstract

Several critical clinical and ethical issues, including immediate treatment decisions, emerged in a case of a double suicide attempt by an elderly couple with a suicide pact and existing do-not-resuscitate (DNR) documentation. This case was complicated by the advanced age of both patients and their family’s expectations and perception of mental illness in the geriatric population. In addition to the myriad of legal and ethical challenges that frequent the end-of-life care, the emerging trend of suicide pacts among the elderly, particularly with existing DNR documentation, warrants further exploration.

## Introduction

A suicide pact is a term describing the decision of two or more people to end their lives together typically at the same time and the same place. While rare and representing only 1% of all suicide cases according to a major epidemiologic study based in England and Wales from 1988 to 1992, suicide pacts are still of great concern [1]. Of note, there is currently no recent data regarding the prevalence or incidence of suicide pacts in the United States. However, those involved typically are people well known to each other such as spouses, families, and friends and of older age and higher social class compared to those who commit suicide alone [2]. Additionally, suicide notes are typically left behind, and in the United States, involve guns or alcohol [2,3]. Of concern, those involved are more likely to include male-female pairs where one is typically dominant to the other, with the dominant partner more commonly associated with a psychiatric diagnosis of depression [1,4].

Further complicating the situation, those involved in suicide and/or suicide pacts may have preexisting do-not-resuscitate (DNR) documentation. Current laws do not comment on those who may use DNR to ensure completion of suicide, which leaves physicians unsure of how to act [4]. Without a doubt, there are contrasting ethical views on whether a DNR order should be honored in those who have attempted suicide, which presents the challenge of honoring autonomy versus beneficence [5]. To further complicate the scenario, family members with uninformed expectations often confound the clinical decision-making. A case report exemplifying many of the above complexities is presented in this study.

## Case presentation

A 93-year-old man developed advanced dementia and moved into a memory unit at an assisted living facility (ALF) with his 92-year-old wife. The man suffered from severe depression and convinced his wife to attempt suicide with him. The wife brought medications and a knife to her husband’s room and they both attempted suicide by overdosing and cutting their wrists. Fortunately, both survived and were admitted to the hospital. After medical stabilization, the wife reported that she had not wanted to die but went along with her husband’s desire to die together. Hospice was consulted for the husband and he was found to not meet the criteria for hospice care as he did not have a terminal illness or a life expectancy of less than six months.

The husband was then admitted to an inpatient psychiatric unit as a patient. He was deemed to not have the capacity to make his own medical decisions and his grandson was appointed as the medical decision-maker. To note, the patient had a previously documented DNR code status after a past suicide attempt, though he did not have any current terminal illnesses. His family initially requested no life-sustaining services be provided to the patient, including withholding food and water, because of the patient’s desire to die.

The medical team felt ethically conflicted because the patient did not have a terminal illness and, with proper medical and psychiatric treatments, was likely to return to his nursing facility once stabilized. After lengthy discussions with multidisciplinary treatment teams and with the patient’s family regarding the nature of the patient’s depression and the numerous treatment options available, the patient’s grandson eventually consented for depression treatment with psychotropics. Subsequently, the patient’s depressive signs and symptoms improved, and he was discharged to his ALF without complications.

## Discussion

Several ethical challenges are routinely considered when addressing DNR status in a patient who attempts suicide. First, overriding a DNR can be considered battery; medical providers often have considerations to avoid having legal actions be made against them [5,6]. Medically assisted suicide is legal only in select states, which also differs from passive assistance where a physician does nothing to prevent suicide. Passive assistance is often equivalent to honoring a DNR [6]. Additionally, overriding a DNR goes against a patient’s autonomy given that the patient has had forethought in creating an advanced directive. Thus, considerations should be made if the patient truly has medical decision-making capacity and autonomy at the time of the suicide attempt as suicidal ideation is often transient and associated with a mental disorder [5]. Beneficence, which supports medical care to those suffering from a treatable disease, also becomes an important factor to consider. With proper treatment and in the setting of an individual without terminal illness, the desire to die can be treated and extinguished. Findings from an analysis of people who died in suicide pacts in England and Wales reveal that improved management of illness, both mental and medical disorders, would likely help avoid suicide pacts [2]. This is exemplified in our case, which also brings to attention the importance of considering the partner’s wellbeing, as the wife in our case did not wish to die but rather acted to appease her husband. A case regarding a double suicide in Croatia in which one of the members involved also experienced depression supports the importance of raising more awareness about the need for a more thoughtful analysis of the decision-making process of a suicide pact itself [7], as the husband in our case is the initiator while the wife is the dependent, and the decision to commit suicide was not evenly shared.

## Conclusions

Current literature regarding suicide pacts with DNR involvement is limited. This case report helps contribute to raising awareness about the ethical dilemma of considering autonomy versus beneficence in the setting of geriatric patients with concerning mood dysregulation and DNR status following a suicide attempt. It also brings to attention the importance of the recognition that a person’s decision-making capabilities can be influenced and affected by his or her partner’s and/or family’s mental health and preconceptions about mental illness, and that a more thoughtful analysis of whether the decision for a suicide pact is independent for all members involved. Although the incidences of elderly suicide pact with DNR are rare at present, these cases are likely to increase due to the steady increase in the aging population, and it is expected to become an emerging issue in the near future for geriatric care.
